# Coordinated Plasticity between Barrel Cortical Glutamatergic and GABAergic Neurons during Associative Memory

**DOI:** 10.1155/2016/5648390

**Published:** 2016-12-14

**Authors:** Fenxia Yan, Zilong Gao, Pin Chen, Li Huang, Dangui Wang, Na Chen, Ruixiang Wu, Jing Feng, Shan Cui, Wei Lu, Jin-Hui Wang

**Affiliations:** ^1^Department of Pathophysiology, Bengbu Medical College, Anhui 233000, China; ^2^State Key Laboratory of Brain and Cognitive Science, Institute of Biophysics, University of Chinese Academy of Sciences, Beijing 100101, China; ^3^School of Pharmacy, Qingdao University, 38 Dengzhou, Shandong 266021, China

## Abstract

Neural plasticity is associated with memory formation. The coordinated refinement and interaction between cortical glutamatergic and GABAergic neurons remain elusive in associative memory, which we examine in a mouse model of associative learning. In the mice that show odorant-induced whisker motion after pairing whisker and odor stimulations, the barrel cortical glutamatergic and GABAergic neurons are recruited to encode the newly learnt odor signal alongside the innate whisker signal. These glutamatergic neurons are functionally upregulated, and GABAergic neurons are refined in a homeostatic manner. The mutual innervations between these glutamatergic and GABAergic neurons are upregulated. The analyses by high throughput sequencing show that certain microRNAs related to regulating synapses and neurons are involved in this cross-modal reflex. Thus, the coactivation of the sensory cortices through epigenetic processes recruits their glutamatergic and GABAergic neurons to be the associative memory cells as well as drive their coordinated refinements toward the optimal state for the storage of the associated signals.

## 1. Introduction

Associative learning is a common approach for information acquisition, and associative memory is presumably essential for the cognitions [1–4]. Cellular mechanism for memory formation is accompanied by neural plasticity [5–9]. For instance, the functional and structural refinements at the neurons and synapses occur in associative memory [10–27]. Memory cells that encode the newly acquired signal and innate signal are recruited after associative learning in the sensory cortices [28, 29] and the downstream brain areas [30, 31]. As interactive balances between excitatory and inhibitory neurons are essential for programming brain codes to manage the well-organized cognitions [32], how these recruited glutamatergic and GABAergic neurons are refined coordinately for the storage of the associated signals remains to be addressed in the mammalians [33, 34].

In conditioned reflex, a model of associative learning and memory, the behaviors in response to unconditioned signals can be induced by conditioned signals [3, 35, 36]. In this cross-modal reflex, the retrieval of the unconditioned signals is induced by the conditioned signals, and the brain areas that encode the unconditioned signals may become processing the conditioned signals. Current reports indicate that paired-stimulations to mouse whiskers and olfaction lead to odorant-induced whisker motion and that the barrel cortical neurons become encoding odor signal alongside whisker signal [28, 29, 37, 38]. As the barrel cortex naturally encodes whisker signal, that is, odor signal is new to the barrel cortex before associative learning, this model should be useful to investigate cellular and molecular mechanisms underlying the storage and retrieval of the newly learnt information. As the associated signals are encoded in the sensory cortex [28], the sensory cortices are the primary locations for the storage of the associated signals, whose plasticity in associative memory has to be elucidated.

To the issues above, we investigated the coordinated recruitments and refinements between barrel cortical excitatory and inhibitory neurons in our mouse model of cross-modal memory. To read out cell-specific mechanisms, glutamatergic neurons were genetically labeled by yellow fluorescent protein, and GABAergic neurons were labeled by green fluorescent protein in the mice [39]. Two-photon cell Ca^2+^ imaging in vivo was used to study whether barrel cortical glutamatergic and GABAergic neurons are recruited to encode the associated signals. Confocal cell imaging and electrophysiology were used to analyze the refinements at these synapses and neurons.

## 2. Methods and Materials

All experiments were performed in accordance with the guidelines by the Administration Office of Laboratory Animals at Beijing, China. All experiment protocols were approved by Institutional Animal Care Unit Committee in the Administration Office of Laboratory Animals at Beijing, China (B10831).

### 2.1. Mouse Model of Associative Memory

To analyze cell-specific mechanism for associative memory we used C57 Thy1-YFP/GAD67-GFP mice [39] whose glutamatergic neurons were genetically labeled by yellow fluorescent protein (YFP) and GABAergic neurons were labeled by green fluorescent protein (GFP).

Two groups of mice in postnatal days 20 were trained by the simultaneous pairing of mechanical whisker stimulus (WS) with odor stimulus (OS, butyl acetate toward the noses) and the unpairing of these stimuli (control), respectively [28]. The paired and unpaired WS and OS were given by multiple-sensory modal stimulator (MSMS, our pattern 201410499466), in which the intensities, time, and intervals of OS and WS were precisely set. OS intensity was sufficient to induce the response of olfactory bulb neurons seen by two-photon Ca^2+^ imaging. WS intensity was sufficient to evoke whisker fluctuation after WS ended. Each of the mice was trained twenty seconds in each time, five times per day with two hours of intervals for ten days. During the training, each mouse was placed in a home-made cage. Cares were taken including no stressful experiment condition and circadian disturbance to the mice that showed normal whisking and symmetric whiskers (please refer to the descriptions and video one in [28] for the details).

Whisker motion tracks were monitored by a digital video camera (50 Hz) and were quantified in retraction time and whisking frequency (MB-Ruler, version 5.0 by Markus Bader, MB-Software Solution, Germany). The responses of mouse whiskers to the odor-test (butyl acetate, 20 sec) were measured before the training and at the end of each training day to quantify the onset time and levels of conditioned reflex (CR). CR-formation was defined to meet the following criteria. The patterns of odorant-induced whisker motion were similar to those of WS-induced whisker motion. Whisking frequency and whisker retraction time significantly increased, compared to control and before the training. As this type of whisker motion induced by odorant was originally induced by WS, the odor signal evoked a recall of whisker signal and then led to whisker motion (please refer to the description and video two in [28] for the details).

The long whiskers (such as arcs 1~2) on the same side and rows were assigned for the mechanical stimulations and for the observations during the odor-test. This selection was based on the studies of cross-modal plasticity [40, 41]. We did not trim the short whiskers since whisker trimming elevated the excitability of the barrel cortex [39].

### 2.2. Fluorescence Labeling

The mice with CR-formation and unpaired control were anesthetized by intraperitoneal injections of urethane (1.5 g/kg). In the surgical operations, the anesthetic depth was lack of reflexes in pinch withdrawal and eyelid blinking. The body temperature was maintained by computer-controlled heating blanket at 37°C. Ca^2+^-sensitive dye, Oregon Green BAPTA-1-AM (OGB-1, Invitrogen, USA), was used to measure neuronal activities. A craniotomy (2 mm) was made on the skull above barrel cortical areas (1 mm posterior to the bregma and 3.5 mm lateral to the midline; [42]). The dura was intact in all experiments. The detailed information about surgical operation, dye loading, and posttreatment for imaging can be referred in our studies [28, 43].

### 2.3. Two-Photon Cell Imaging

The anesthetic depth for doing two-photon cell imaging in vivo of the control and CR-formation mice was set at the moderate reflexes in pinch withdrawal and eyelid blinking, as well as the responses of their whiskers to the test stimuli [28, 43]. Ca^2+^ imaging was done one hour after dye injections under a two-photon microscope (Olympus FV-1000, Tokyo, Japan), which was equipped by the two-photon laser-beam generator (Mai Tai, Physical Spectrum, USA) and mounted to an upright microscope (Olympus BX61WI) with water immersion objectives (IR-LUMPLan Fl, 40x, 0.8NA). A wave of two-photon laser beam at 810 nm was given to excite OGB and examine neuronal responses to WS and OS. The emission wavelength was 525 nm for Ca^2+^-binding OGB. Subsequently, the neuron identities were tested by giving a wave at 920 nm that excited GFP that labeled GABAergic neurons and YFP that labeled glutamatergic neurons. Although the peaks of optical emission spectra are closely for GFP (510 nm) and YFP (525 nm), the separation of their images can be done by setting optical gratings with different windows for their unmixing (please see the identifications of GFP and YFP in Section 2.7). Average power delivered to the cortices was less than 75 mW to minimize photo bleach. Whole field images were acquired at 10 Hz of frame rate (256 × 256 pixels). The parameters for photomultiplier tube (PMT) and laser beam were locked in the measurements throughout all experiments to have the consistence for data comparison.

Similar to the stimuli in behavioral task, an odor-test pulse toward the noses or mechanical pulses to the whiskers on the contralateral side of two-photon imaged cortices were used to induce cell responses. Stimulus patterns were pair-pulses (OS versus WS or WS versus OS) and pulse intervals were 60 seconds [28].

### 2.4. Imaging Data Analyses

Cellular Ca^2+^ fluorescence signals in response to stimuli were acquired by Fluoview-10 software (Olympus Inc., Japan) and analyzed in cell bodies by NIH ImageJ and MATLAB (MathWorks). To reduce photon and PMT noise, a median filter (radius, 1 pixel) was used to all images. Ca^2+^ signals were normalized and presented as relative fluorescence changes (Δ*F*/*F*). Basal fluorescence (*F*) was an averaged value before stimuli, and Δ*F* values were the differences between Ca^2+^ signals from evoked response and basal fluorescence [43]. All fluorescence signals were subtracted from the noise signals of unstained blood vessels. Normalized Ca^2+^ signals were smoothed by a low-pass Butterworth filter to remove low-level fluctuation and to minimize distortions from Ca^2+^ transient [44]. It is noteworthy that fluorescent signals are measured at a time, but not repetitively, in order to prevent their photo bleach and effects on signal strength analyses. The effective signals from each of active cells were judged based on a criterion that their relative fluorescence changes were greater than 2.5 times of standard deviation of baseline values lasting for 500 ms [28]. The magnitudes of Ca^2+^ transient signals, that is, activity strengths, were measured from the point at 2.5 times of standard deviation of baseline values to their peaks. The durations of Ca^2+^ transient signals were measured as the time from the point at 2.5-fold of standard deviation of baseline values in the rising phase to the point at 2.5-fold of standard deviation of baseline values in the decay phase [28, 43].

### 2.5. Brain Slices and Neurons

Cortical slices (400 *μ*m) were prepared from the mice of CR-formation and unpaired controls. They were anesthetized by inhaling isoflurane and decapitated by a guillotine. The slices were cut by vibratome in the oxygenated (95% O_2_/5% CO_2_) artificial cerebrospinal fluid (ACSF), in which the chemical concentrations (mM) were 124 NaCl, 3 KCl, 1.2 NaH_2_PO_4_, 26 NaHCO_3_, 0.5 CaCl_2_, 4 MgSO_4_, 10 dextrose, and 5 HEPES, pH 7.35 at 4°C. The slices were held in the oxygenated ACSF (124 NaCl, 3 KCl, 1.2 NaH_2_PO_4_, 26 NaHCO_3_, 2.4 CaCl_2_, 1.3 MgSO_4_, 10 dextrose, and 5 HEPES, pH 7.35) at 25°C for 2 hours. The slices were transferred to submersion chamber (Warner RC-26G) that was perfused with the oxygenated ACSF at 31°C for whole-cell recording [45].

Electrophysiological recordings on the neurons in layers II-III of the barrel cortex were conducted under DIC-fluorescent microscope (Nikon FN-E600, Japan). The wavelength at 488 nm excited GFP, and the wavelength at 575 nm excited YFP. GABAergic neurons showed basket shape and fast spiking with less adaptation in spike amplitudes and frequency [46–48]. Glutamatergic neurons showed pyramidal shape and regular spikes with the adaptation of spike amplitudes and frequency [49]. Cerebral slices were coronal sections including the barrels correspondent to the projection from long whiskers that were stimulated in pairing WS and OS training.

### 2.6. Whole-Cell Recording

Cortical neurons were recorded by MultiClamp-700B amplifier in voltage-clamp for their synaptic activities. Electrical signals were inputted into pClamp-10 (Axon Instrument Inc., CA, USA) for data acquisition and analyses. Output bandwidth in this amplifier was 3 kHz. The pipette solution for studying excitatory synapses included (mM) 150 K-gluconate, 5 NaCl, 5 HEPES, 0.4 EGTA, 4 Mg-ATP, 0.5 Tris-GTP, and 5 phosphocreatine (pH 7.35; [50, 51]). The solution for studying inhibitory synapses contained (mM) 130 K-gluconate, 20 KCl, 5 NaCl, 5 HEPES, 0.5 EGTA, 4 Mg-ATP, 0.5 Tris-GTP, and 5 phosphocreatine [52]. Pipette solutions were freshly made and filtered (0.1 *μ*m), osmolarity was 295~305 mOsmol, and pipette resistance was 5~6 MΩ.

The functions of GABAergic neurons were assessed based on their active intrinsic properties and inhibitory outputs [53]. The function status of their inhibitory outputs was evaluated by recording spontaneous inhibitory postsynaptic currents (sIPSCs) under the voltage-clamp on glutamatergic neurons in the presence of 10 *μ*M 6-cyano-7-nitroquinoxaline-2,3-(1H,4H)-dione (CNQX) and 40 *μ*M D-amino-5-phosphonovanolenic acid (D-AP5) in ACSF to block ionotropic glutamate receptors [54]. 10 *μ*M bicuculline was washed onto the slices at the end of experiments to test whether synaptic responses were mediated by GABA_A_R, which blocked sIPSCs in our experiments. The series and input resistance in all of the neurons were monitored by injecting hyperpolarization pulses (5 mV/50 ms) and calculated by voltage pulses versus instantaneous and steady-state currents. It is noteworthy that pipette solution with the high concentration of chloride ions makes the reversal potential to be −42 mV. sIPSCs will be inward when the membrane holding potential is at −65 [52, 54].

The functions of glutamatergic neurons were assessed based on their active intrinsic property and excitatory outputs [53]. The function status of their excitatory outputs was evaluated by recording spontaneous excitatory postsynaptic currents (sEPSCs) on GABAergic or glutamatergic neurons in presence of 10 *μ*M bicuculline in ACSF to block ionotropic GABA receptors [53]. 10 *μ*M CNQX and 40 *μ*M DAP-5 were added into ACSF perfused onto the slices at the end of experiments to examine whether synaptic responses were mediated by GluR, which blocked EPSCs in our experiments. The series and input resistance for all of the cells were monitored by injecting hyperpolarization pulses (5 mV/50 ms) and calculated by voltage pulses versus instantaneous and steady-state currents.

Action potentials at these cortical neurons were induced by injecting depolarization pulses, whose intensity and duration were altered based on the aim of the experiments. The ability to convert excitatory inputs into digital spikes was evaluated by interspike intervals (ISI) when depolarization pulses (200 ms in the duration and the thresholds for 10 ms pulse-induced spike in the intensity) were given, as well as by input-outputs (spikes versus normalized stimuli) when various stimuli were given [55]. Neuronal intrinsic properties, which were used to evaluate the neuronal excitability in our study, included spike threshold potentials (Vts) and absolute refractory period (ARP). We did not measure the rheobase to show neuronal excitability, since this strength-duration relationship was used to indicate the ability to fire a single spike. Our study was to measure the ability of producing sequential spikes. Vts were the voltages of producing spikes [56–58]. ARPs were measured by injecting paired-depolarization pulses (3 ms) into these neurons after each spike. By changing interpulse intervals, we defined ARPs as the time from a complete spike to its subsequent spike at 50% probability [59, 60].

The recording of spontaneous synaptic currents, instead of the evoked synaptic currents, is based on the following reasons. sEPSC and sIPSC amplitudes represent the responsiveness and the densities of postsynaptic receptors. The frequencies imply the probability of transmitter release from an axon terminal and the number of presynaptic axons innervated on the recorded neuron [61, 62]. These parameters can be used to analyze presynaptic and postsynaptic mechanisms about the neuronal interaction. The evoked postsynaptic currents cannot separate these mechanisms. We did not add TTX in the ACSF to record miniature postsynaptic currents as we had to record neuronal excitability. As the frequency of synaptic activities was less than those of sequential spikes and the spontaneous spikes were never recorded on the neurons in our cortical slices, sIPSCs and sEPSCs were not generated from spontaneous action potential. The synaptic events in our recording are presumably miniature postsynaptic currents. This point is granted by a single peak of postsynaptic currents in our studies [49, 63].

Data were analyzed if the recorded neurons had the resting membrane potentials negatively more than −60 mV and action potential amplitudes more than 90 mV. The criteria for the acceptance of each experiment also included less than 5% changes in resting membrane potential, spike magnitude, and input resistance throughout each experiment. Input resistance was monitored by measuring cellular responses to hyperpolarization pulse at the same values as the depolarization that evoked action potentials. To estimate the effects of associative learning on the neuronal spikes and synaptic transmission, we measured sEPSC, sIPSC, ISI, ARP, and Vts under the conditions of control and associative memory, which were presented as mean ± SE. The comparisons of these data before and after associative learning were done by *t*-test.

### 2.7. Cellular Morphological Imaging in the Barrel Cortices

The control and CR-formation mice were anesthetized by intraperitoneal injections of sodium pentobarbital and perfused by 4% paraformaldehyde in 0.1 M phosphate buffer solution (PBS) into left ventricle until their bodies were rigid. The brains were quickly isolated and fixed in 4% paraformaldehyde PBS for additional 24 hours. The cerebral brains were sliced in a series of coronal sections at 80 *μ*m, which included the barrels correspondent to the projection from long whiskers that were stimulated in pairing WS and OS training. These slices were rinsed by PBS for three times, air-dried, and cover-slipped. The images for YFP-labeled glutamatergic neurons and GFP-GABAergic neurons in cortical layers II~III were photographed under the confocal microscopy with oil lens (Plan Apo VC 60x, 1.4NA; Nikon A1R plus, Tokyo, Japan). The excited wavelength was 488 nm for GFP and YFP. Although the peaks of GFP and YFP emission wavelengths are closely at 510 and 525 nm, respectively, we scanned the images of these neurons through setting the optical grating in 505~515 nm for GFP and the optical grating in 545~555 nm for YFP, to separate their fluorescent images. In terms of morphological interactions between glutamatergic and GABAergic neurons in mouse barrel cortex, the mutual innervations between these cells were measured by counting the contacts of presynaptic boutons with postsynaptic neurons and dendritic spines. These quantifications were done by ImageJ (version 1.47; National Institute of Health, USA). The analyses of the processes and spines were given in the method of our previous study [39].

### 2.8. An Analysis of MicroRNA by MicroRNA-Sequencing

Total RNAs were isolated from the dissected samples from the barrel cortices of control and CR-formation mice, which were done by TRIzol reagent (Life Technologies, Grand Island, NY, USA) based on manufacturer's guidelines. The fractionation and preparation of smaller RNAs (18–30 nts) for high throughput sequencing were done by using the Protocol of Small RNA Sample Preparation (Illumina). The sequencing of low molecular weight RNA was done by using Illumina HiSeq 2000 system (Illumina, CA, USA).

Small RNAs in HiSeq deep sequencing included miRNA, siRNA, piRNA, rRNA, tRNA, snRNA, snoRNA, repeat associated sRNA, and degraded tags in the exons or introns. By comparing our data with those in the databases and picking out the overlap on genome location between our data and the databases, small RNAs were annotated into different categories. Those which could not be annotated would be used to predict novel miRNA by the self-developed software Mireap (BGI-Shenzhen, China).

50 nt sequence tags from HiSeq sequencing went through the data cleaning analysis. This analysis would get rid of low quality tags and 5′ adaptor contaminants from 50 nt tags in order to get credible clean tags. Then, the length distributions of these clean tags as well as the common and specific sequences in the samples between control and CR-formation were summarized. Finally, the standard analysis annotated the clean tags into the different categories and took those which could not be annotated to any category to predict novel miRNA and potential known miRNA. With miRNA data, the target prediction for miRNA and the GO enrichment and the KEGG pathway for target genes were analyzed [63, 64].

### 2.9. Statistical Analyses

The paired *t*-test was used in the comparisons of the experimental data before and after associative learning, before and after bicuculline application, and the neuronal responses to whisker stimulus and odorant stimulus in each of the mice. One-way ANOVA was applied to make the statistical comparisons in the changes of neuronal activities and morphology between control and CR-formation groups.

## 3. Results

### 3.1. Barrel Cortical Glutamatergic and GABAergic Neurons Become Encoding Odor and Whisker Signals

After associative learning by pairing odor stimuli (OS) and whisker stimuli (WS) simultaneously, the mouse whiskers became responding to OS (odorant-induced whisker motion) alongside WS (whisker-induced whisker motion). Whisking frequency and retraction duration in response to OS rise, compared to the whisking before training and in unpaired control (Figure S1 in Supplementary Material available online at http://dx.doi.org/10.1155/2016/5648390). As the patterns of odorant-induced and whisker-induced whisker motions are similar [28], the odor signal may induce a recall of the whisker signal, that is, associative memory. Moreover, the inhibition of the barrel cortex removes this cross-modal reflex, such that associative learning enables this cortical area to receive and process the odor signal for its storage and retrieval [28].

**Figure 1:**
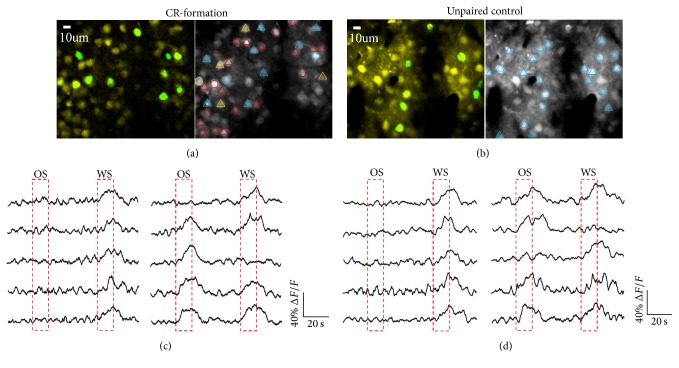
Glutamatergic and GABAergic neurons in the barrel cortex respond to the odor signal (OS) and whisker signal (WS) after their pairing. Cellular activities were detected by imaging Ca^2+^ signals under a two-photon microscope in control (*n* = 7) and CR-formation mice (*n* = 7), in which glutamatergic neurons were genetically labeled by YFP and GABAergic neurons were labeled by GFP. (a) Left panel shows the images of the glutamatergic neurons (yellow) and GABAergic neurons (green) in the barrel cortex from a CR-formation mouse. Right panel shows the neurons in response to WS and/or OS, which are defined as Δ*F* larger than 2.5-fold of standard deviation of baseline values. The neurons labeled by red are WS/OS-responsive cells. The neurons labeled by blue respond to WS. The neurons labeled by yellow respond to OS. (b) Left panel shows the images of glutamatergic neurons (yellow) and GABAergic neurons (green) in the barrel cortex from an unpaired control mouse. Right panel illustrates the neurons labeled by blue in response to WS. In (a)~(b), the glutamatergic neurons are marked as triangles and GABAergic neurons are marked as circles. (c) shows the digitized Ca^2+^ signals recorded from glutamatergic neurons in response to WS versus OS from unpaired control mouse (left panel) and CR-formation mouse (right). (d) shows the digitized Ca^2+^ signals recorded from GABAergic neurons in response to WS versus OS from an unpaired control mouse (left panel) and CR-formation mouse (right). The calibration bars are 40% Δ*F*/*F* and 20 seconds.

Whether barrel cortical neurons encoded whisker and odor signals was examined by two-photon Ca^2+^ imaging in the mice with conditioned reflexes (CR) and unpaired controls. Their glutamatergic and GABAergic neurons were identified based on their genetical labeling by different fluorescent proteins [39]. Barrel cortical neurons appear to encode both odorant and whisker signals after associative learning. As showed in Figure 1, some neurons respond to WS and OS (yellow-labeled), while others respond to WS (green) or OS only (red).  Figure 1(b) shows the digital Ca^2+^ signals of glutamatergic neurons in response to WS and OS from CR-formation mice (right panels) versus controls (lefts).  Figure 1(c) shows the digitized Ca^2+^ signals of GABAergic neurons in response to WS and OS in CR-formation mice (right panels) versus controls (left). Therefore, barrel cortical glutamatergic and GABAergic neurons are recruited as associative memory cells that encode odor signal alongside whisker signal.

### 3.2. Barrel Cortical Glutamatergic Neurons Are Upregulated after Associative Learning

The recruitments of glutamatergic neurons to be associative memory cells may be caused by the upregulation of excitatory synaptic inputs and coding ability as well as the downregulation of inhibitory synaptic inputs, which we examined at YFP-labeled barrel cortical glutamatergic neurons in layers II~III of the barrel cortices from CR-formation mice versus controls. The morphological changes of their spines were analyzed on apical dendrites. Spontaneous excitatory postsynaptic currents (sEPSCs) were recorded to assess excitatory synaptic activities. The capability to encode spikes was measured to estimate active intrinsic property. Spontaneous inhibitory postsynaptic currents (IPSCs) were recorded to assess inhibitory synaptic function [39].

**Figure 2:**
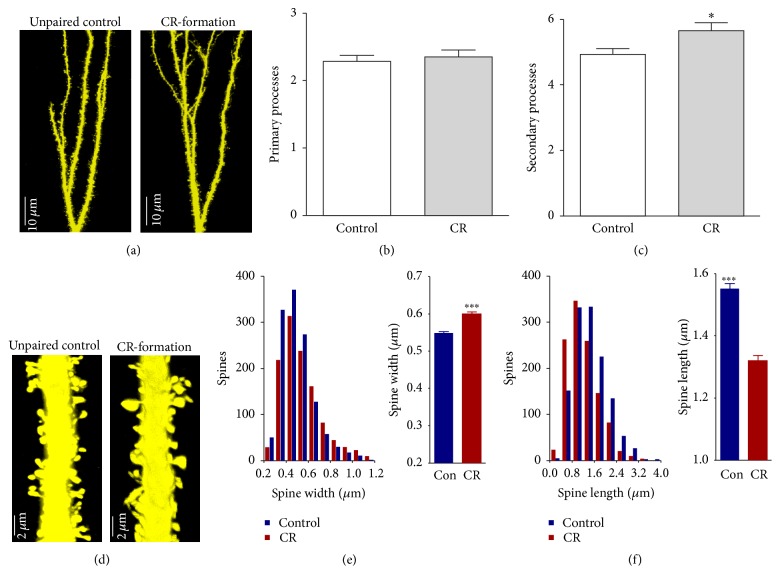
The number of dendrites and the head of the spines on barrel cortical glutamatergic neurons are upregulated after associative leaning. (a) shows the branches of the secondary processes appear denser in CR-formation mouse (right panel) than controls (left). (b) shows the comparisons of primary processes per apical dendrite in CR-formation (gray bar, *n* = 20 neurons) and controls (white; *p* = 0.3, *n* = 25). (c) shows the comparisons of the secondary processes per apical dendrite in CR-formation (gray bar, *n* = 20 neurons) and controls (white; asterisk, *p* < 0.05, *n* = 25). (d) The spine volume appears larger and the spine length is shorter on CR-formation neurons (right panel) than controls (left). (e)~(f) show the comparisons of spine widths (e) and lengths (f) from CR-formations (red bar, *n* = 1162) and controls (Con; blue bar, *n* = 1273). The spine head tends to be large and the spine length tends to be short (three asterisks, *p* < 0.001).

The branches of the secondary processes appear denser in CR-formation (right panel in Figure 2(a)) than controls (left). Although there is no alternation in the primary processes (Figure 2(b)), the secondary processes per apical dendrite are higher in CR-formation neurons (5.65 ± 0.24, *n* = 20 neurons) than controls (4.92 ± 0.2, *n* = 25, *p* = 0.02; Figure 2(c)). In terms of spine morphology, we measured head width and length since large head and short neck are presumably functional spines that form the synapses with axon bouton [65, 66]. After associative learning, the spine head appears larger and the spine length appears shorter in CR-formation neurons (right panel in Figure 2(d)) than controls (left). The spine widths are 0.6 ± 0.006 *μ*m in CR-formation (red bar in Figure 2(e)) and 0.55 ± 0.004 *μ*m in control (blue). The spine lengths are 1.32 ± 0.016 *μ*m in CR-formations (red bar in Figure 2(f)) and 1.55 ± 0.017 *μ*m in controls (blue). The spine head tends to be larger and the spine length tends to be shorter (*p* < 0.001, *n* = 1162 for CR-formation and 1273 for control). Associative learning makes the dendritic spines on the glutamatergic neurons being enlarged for synapse formation, which is consistent with the suggestion that the enlarged spines play a role in the memory and cognition [66].

**Figure 3:**
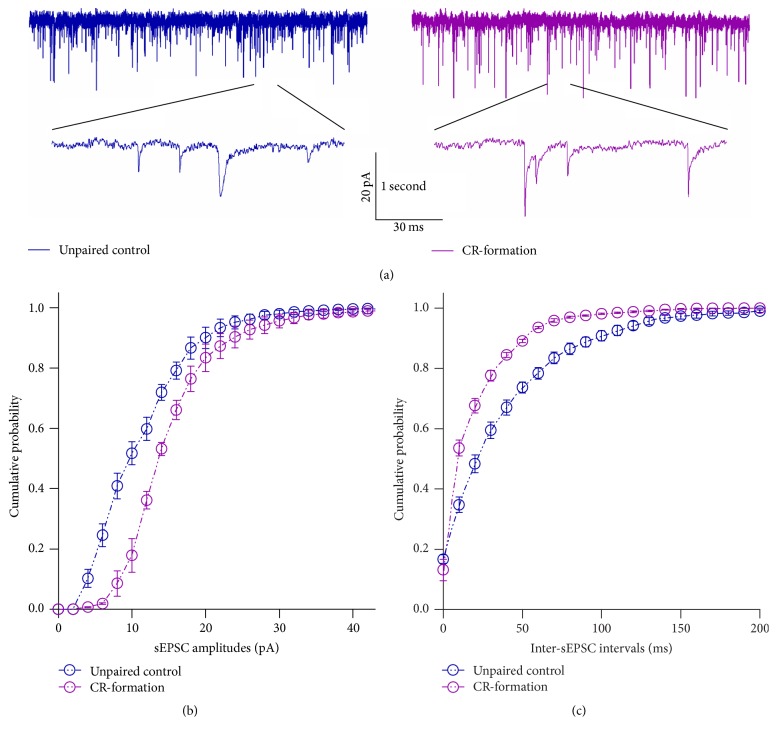
Excitatory synaptic transmission on barrel cortical pyramidal neurons increases after pairing WS and OS. Spontaneous excitatory postsynaptic currents (sEPSCs) were recorded on the pyramidal neurons in cortical slices under voltage-clamp (holding potential at −70 mV) in presence of 10 *μ*M bicuculline. (a) shows sEPSCs recorded on the neurons in control (dark-blue trace in left panel) and CR-formation (dark-red in right). Bottom traces are the expanded waveforms selected from top traces. Calibration bars are 20 pA, 1 second (top) and 30 ms (bottom). (b) shows cumulative probability versus sEPSC amplitudes from control (dark-blue symbols, *n* = 16) and CR-formation neurons (dark-red symbols, *n* = 15). (c) illustrates cumulative probability versus inter-sEPSC intervals from control (dark-blue symbols, *n* = 16) and CR-formation (dark-red symbols, *n* = 15).

Figure 3 illustrates the effects of associative learning on excitatory synaptic transmission in barrel cortical glutamatergic neurons. sEPSCs after associative learning appear higher, compared to the controls (Figure 3(a)).  Figure 3(b) shows cumulative probability versus sEPSC amplitudes in CR-formation neurons (*n* = 15) and controls (*n* = 16).  Figure 3(c) shows cumulative probability versus inter-sEPSC intervals from CR-formation neurons (*n* = 15) and controls (*n* = 16). Statistical analysis indicates that sEPSC amplitudes and frequency (i.e., 1/inter-sEPSC interval) are higher in CR-formation than control (*p* < 0.01). Associative learning upregulates excitatory synaptic transmission in barrel cortical glutamatergic neurons.

**Figure 4:**
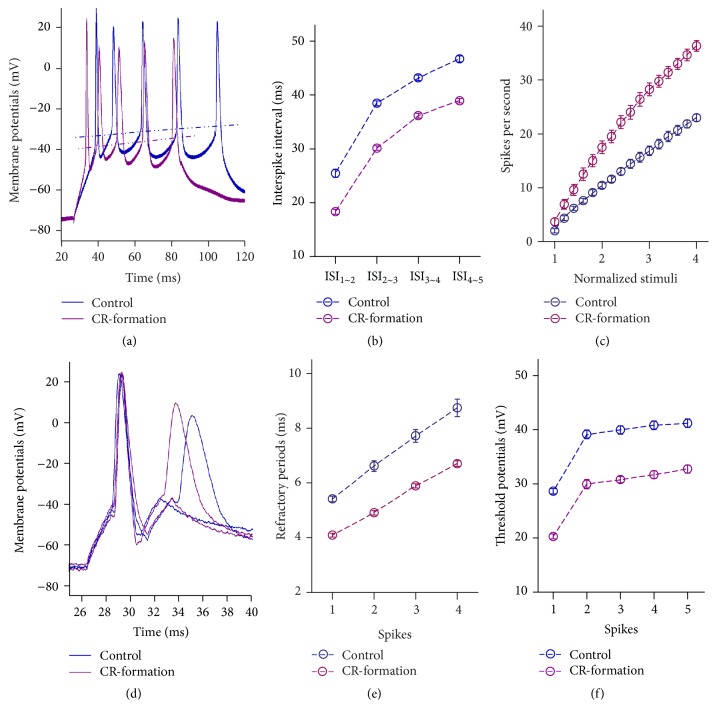
The capability to encode spikes on barrel cortical pyramidal neurons increases after pairing WS and OS. The spikes were induced by depolarization pulse under voltage-clamp recording on glutamatergic neurons in cortical slices. (a) Traces illustrate depolarization-induced spikes on the neurons from control (blue trace) and CR-formation (dark-red). (b) shows interspike intervals for spikes 1~2 to spikes 4~5 from controls (dark-blue symbols, *n* = 21) and CR-formations (dark-red symbols, *n* = 20). (c) shows spikes per second versus normalized stimuli from control (dark-blue symbols, *n* = 21) and CR-formation (dark-red symbols, *n* = 20). (d) Traces show the measurements of spike refractory periods on the neurons from controls (dark-blue trace) and CR-formations (dark-red). (e) shows refractory periods versus spikes 1 to 4 from controls (dark-blue symbols, *n* = 21) and CR-formations (dark-red symbols, *n* = 20). (f) shows the threshold potential versus spikes 1 to 5 from controls (dark-blue symbols, *n* = 21) and CR-formations (dark-red symbols, *n* = 20).

Figures 4(a)~4(c) show the ability of glutamatergic neurons to convert excitatory inputs into spikes. The neurons from CR-formation mice have higher ability to encode spikes (dark-red trace in Figure 4(a)), compared to controls (dark-blue).  Figure 4(b) illustrates interspike intervals (ISI) in CR-formation neurons (dark-red symbols) and controls (dark-blue). ISI values for spikes 1~2 to 4~5 are 18.32 ± 0.44, 30.13 ± 0.4, 36.17 ± 0.43, and 38.94 ± 0.4 in CR-formation neurons (*n* = 20) and are 25.46 ± 0.76, 38.46 ± 0.45, 43.19 ± 0.5, and 46.7 ± 0.57 in controls (*n* = 21). The ISI values for corresponding spikes in two sources of neurons are different (*p* < 0.01). Moreover, in spikes versus normalized stimulation (Figure 4(c)), input-output curve for CR-formation neurons (dark-red symbols) shifts left-high, compared with that for the controls (dark-blue). Associative learning upregulates the ability of barrel cortical glutamatergic neurons to convert excitatory inputs into digital spikes.

Figures 4(d)~4(f) show the changes of VGSC-mediated mechanisms, such as spike refractory periods (RPs) and threshold potentials (Vts). Vts (Figure 4(a)) and RPs (Figure 4(d)) appear lower in CR-formation neurons than controls. RP values for spikes 1 to 4 are 4.1 ± 0.07, 4.91 ± 0.08, 5.9 ± 0.08, and 6.7 ± 0.1 in CR-formations (Figure 4(e), *n* = 20) and are 5.4 ± 0.1, 6.62 ± 0.2, 7.72 ± 0.24, and 8.74 ± 0.32 in controls (*n* = 21). Vts values for spikes 1 to 5 are 20.26 ± 0.45, 29.94 ± 0.84, 30.77 ± 0.6, 31.7 ± 0.7, and 32.7 ± 0.75 in CR-formations (Figure 4(f); *n* = 20) and are 20.26 ± 0.45, 39.13 ± 0.8, 39.94 ± 0.77, 40.82 ± 0.8, and 41.19 ± 0.77 in the controls (*n* = 21). RP and Vts for corresponding spikes in CR-formation neurons and controls are different (*p* < 0.01). Associative learning upregulates active intrinsic properties in barrel cortical glutamatergic neurons.

**Figure 5:**
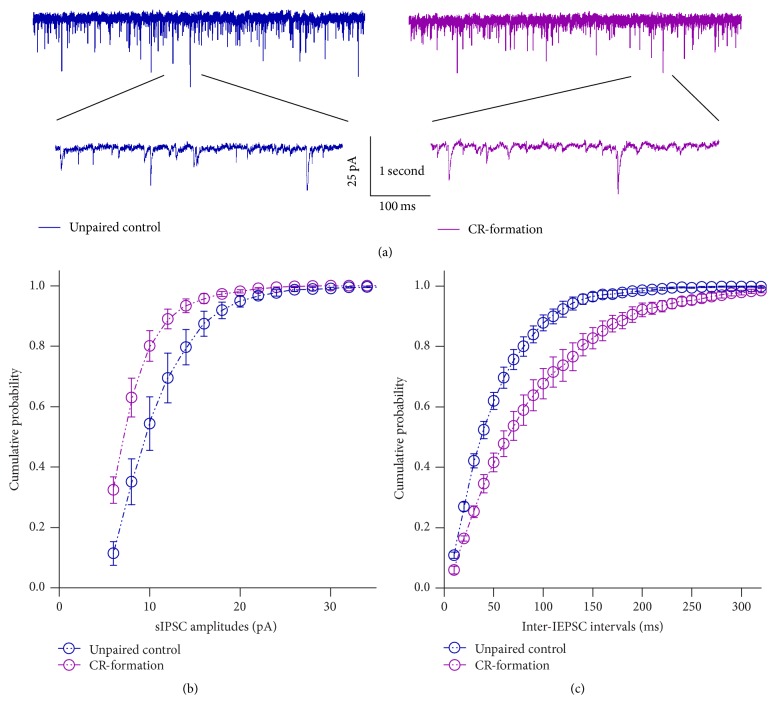
Inhibitory synaptic transmission on barrel cortical pyramidal neurons decreases after pairing WS and OS. Spontaneous inhibitory postsynaptic currents (sIPSCs) were recorded on glutamatergic neurons in cortical slices under voltage-clamp (holding potential at −65 mV) in presence of 10 *μ*M CNQX and 40 *μ*M D-AP5. (a) Traces show sIPSCs recorded on the neurons from controls (dark-blue in left panel) and CR-formation (dark-red in right). The bottom traces present the expanded waveforms selected from top traces. Calibration bars are 25 pA, 1 second (top) and 100 ms (bottom). (b) shows cumulative probability versus sIPSC amplitudes from CR-formation (dark-red symbols, *n* = 12) and control (dark-blue symbols, *n* = 12). (c) shows cumulative probability versus inter-sIPSC intervals from CR-formations (dark-red symbols, *n* = 12) and controls (dark-blue symbols, *n* = 12).

The effect of associative learning on inhibitory synaptic functions in barrel cortical glutamatergic neurons is showed in Figure 5. sIPSCs appear lower in CR-formation neurons than controls (Figure 5(a)).  Figure 5(b) shows cumulative probability versus sIPSC amplitude from CR-formations (*n* = 12) and controls (*n* = 12).  Figure 5(c) shows cumulative probability versus inter-sIPSC intervals in CR-formation (*n* = 12) and controls (*n* = 12). Statistical analyses indicate that sIPSC amplitudes and frequency (1/inter-sIPSC interval) are lower from CR-formation neurons than controls (*p* < 0.01). Associative learning attenuates inhibitory synaptic transmission in barrel cortical glutamatergic neurons.

In summary, the associative learning leads to the upregulation of the spines, excitatory synaptic transmission, and encoding ability as well as the downregulation of GABAergic synaptic transmission on glutamatergic neurons in the barrel cortex. These changes may facilitate the recruitment and refinement of barrel cortical glutamatergic neurons to be associative memory cells. We subsequently studied plasticity at barrel cortical inhibitory neurons after associative learning.

### 3.3. Barrel Cortical GABAergic Neurons Change in Homeostatic Manner after Associative Learning

In terms of plasticity at barrel cortical GABAergic neurons in associative learning, we studied the processes, excitatory synaptic inputs, and active intrinsic property of GFP-labeled GABAergic neurons in CR-formation and control mice. Their branches were counted to merit their receptive fields. sEPSCs were recorded to assess their receiving of excitatory synaptic transmission. The ability of converting excitatory inputs into digital spikes was measured to evaluate their active intrinsic properties [39].

**Figure 6:**
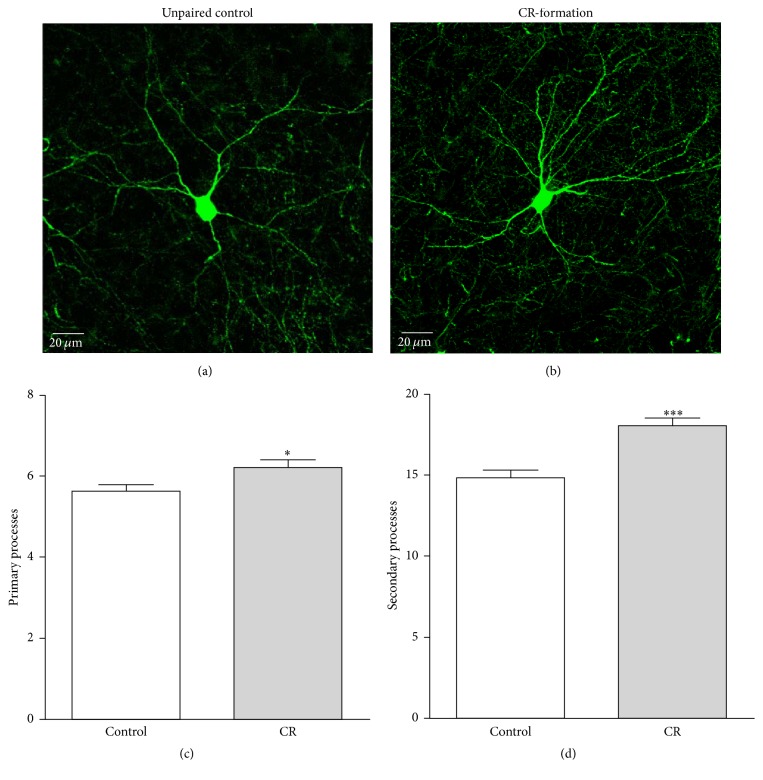
The processes of GABAergic neurons in the barrel cortices increase after pairing WS and OS. (a)~(b) illustrate that process branches appear denser in CR-formations (a) than controls (b). (c) Primary processes per GABAergic neuron are higher in CR-formation mice (gray bar, *n* = 43) than controls (white, *n* = 40; asterisk, *p* < 0.05). (d) The secondary process branches per neuron are higher in CR-formation (gray bar) than control mice (white bar, three asterisks, *p* < 0.001).

The process branches from GFP-labeled GABAergic neurons were counted in CR-formation and control mice. Their process branches appear denser in CR-formation neurons (Figure 6(a)) than in controls (Figure 6(b)). The primary processes per neuron are statistically higher in CR-formations (6.2 ± 0.2, *n* = 43 neurons) than controls (5.63 ± 0.16, *n* = 40; *p* = 0.02; Figure 6(c)). The secondary processes per neuron are higher in CR-formations (18 ± 0.54, *n* = 43 cells) than controls (14.8 ± 0.52, *n* = 40; *p* < 0.001; Figure 6(d)). Because these processes are used to receive synaptic input signal, the GABAergic neurons have high capacity to receive excitatory inputs after associative learning.

**Figure 7:**
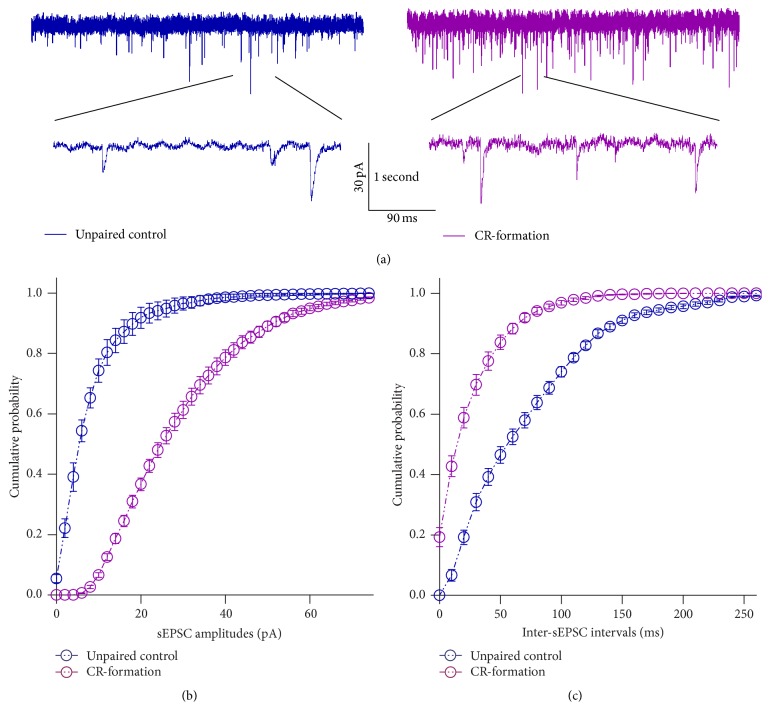
Excitatory synaptic transmission on barrel cortical GABAergic neurons increases after pairing WS and OS. Spontaneous excitatory postsynaptic currents (sEPSCs) were recorded on the GFP-labeled GABAergic neurons in cortical slices under voltage-clamp (holding potential at −65 mV) in presence of 10 *μ*M bicuculline. (a) shows sEPSCs recorded on the neurons from controls (dark-blue in left panel) and CR-formation (dark-red in right). The bottom traces are the expanded waveforms selected from top traces. Calibration bars are 30 pA, 1 second (top) and 90 ms (bottom). (b) shows cumulative probability versus sEPSC amplitudes from control (dark-blue symbols, *n* = 12) and CR-formation (dark-red, *n* = 12). (c) shows cumulative probability versus inter-sEPSC intervals from controls (dark-blue symbols, *n* = 12) and CR-formation (dark-red symbols, *n* = 12).

Figure 7 illustrates the effect of associative learning on excitatory synaptic transmission in barrel cortical GABAergic neurons. sEPSCs appear higher in CR-formation neurons than controls (Figure 7(a)).  Figure 7(b) shows cumulative probability versus sEPSC amplitude from CR-formations (*n* = 12) and control (*n* = 12).  Figure 7(c) shows cumulative probability versus inter-sEPSC intervals from CR-formations (*n* = 12) and controls (*n* = 12). Statistical analysis indicates that sEPSC amplitude and frequency are higher in CR-formation neurons than controls (*p* < 0.01). The barrel cortical GABAergic neurons receive the increased driving force from excitatory neurons after associative learning.

**Figure 8:**
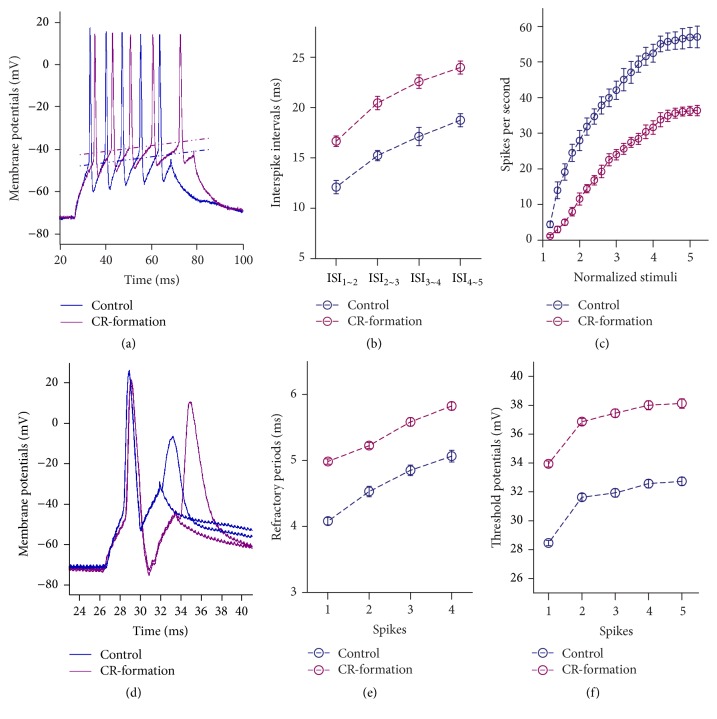
The ability to encode spikes in barrel cortical GABAergic neurons decreases after pairing WS and OS. Spikes were induced by depolarization pulses under voltage-clamp recording on the GABAergic neurons in cortical slices. (a) illustrates depolarization-induced spikes on the neurons from controls (dark-blue trace) and CR-formation (dark-red). (b) shows interspike intervals for spikes 1~2 to spikes 4~5 from controls (dark-blue symbols, *n* = 21) and CR-formation (dark-red symbols, *n* = 22). (c) illustrates spikes per second versus normalized stimuli (input-output) from control (dark-blue symbol, *n* = 21) and CR-formation (dark-red, *n* = 22). (d) shows the measurement of spike refractory periods on the neurons from control (dark-blue trace) and CR-formation (dark-red). (e) shows refractory periods versus spikes 1 to 4 from controls (dark-blue symbols, *n* = 21) and CR-formation mice (dark-red symbols, *n* = 22). (f) illustrates threshold potentials versus spikes 1 to 5 from controls (dark-blue symbols, *n* = 21) and CR-formation (dark-red symbols, *n* = 22).

Figures 8(a)~8(c) show the abilities of GABAergic neurons to convert excitatory inputs into digital spikes. The neurons from CR-formation have lower ability to encode digital spikes, compared to control (Figure 8(a)).  Figure 8(b) shows interspike interval (ISI) in GABAergic neurons from CR-formation mice (dark-red symbols) and control (dark-blue). ISI values for spikes 1~2 to 4~5 are 16.65 ± 0.52, 30.45 ± 0.67, 22.58 ± 0.67, and 24 ± 0.65 in CR-formation neurons (*n* = 22) and are 12.1 ± 0.66, 15.23 ± 0.5, 17.12 ± 0.9, and 18.74 ± 0.65 in controls (*n* = 21). ISI values for corresponding spikes in CR-formation neurons and controls are different (*p* < 0.01). Moreover, Figure 8(c) shows spikes versus normalized stimuli. Input-output curve for CR-formation neurons (dark-red symbols) shifts to right-low, compared to that in controls (dark-blue). Associative learning downregulates the capability of GABAergic neurons to convert excitatory input into digital spikes.

Figures 8(d)~8(f) show VGSC-mediated mechanisms at GABAergic neurons. Vts and RPs (Figures 8(a) and 8(d)) appear higher in CR-formation neuron than control. RP values for spikes 1 to 4 are 4.98 ± 0.04, 5.22 ± 0.05, 5.58 ± 0.06, and 5.82 ± 0.06 in CR-formations (Figure 8(e), *n* = 20) and are 4.1 ± 0.06, 4.52 ± 0.1, 4.85 ± 0.08, and 5.1 ± 0.09 in controls (*n* = 21). The Vts values for spikes 1~5 are 33.94 ± 0.21, 36.87 ± 0.24, 37.45 ± 0.28, 38 ± 0.3, and 38.13 ± 0.33 in CR-formations (Figure 8(f); *n* = 22) and are 28.46 ± 0.2, 31.63 ± 0.26, 31.93 ± 0.3, 32.57 ± 0.28, and 32.73 ± 0.3 in controls (*n* = 21). The RP and Vts values for corresponding spikes in CR-formation neurons and controls are different (*p* < 0.01). Associative learning reduces active intrinsic properties in barrel cortical GABAergic neurons.

In summary, associative learning upregulates excitatory synaptic inputs and downregulates spike-encoding ability in barrel cortical GABAergic neurons, a homeostatic process maintained among different subcellular compartments [57]. The upregulated excitatory inputs facilitate the functional recruitment of GABAergic neurons to be associative memory cells. The downregulation of the encoding ability in GABAergic neurons and their output synapse functions facilitate the recruitment and refinement of glutamatergic neurons in the barrel cortex after associative learning.

### 3.4. Innervation between Glutamatergic and GABAergic Neurons Is Upregulated in Associative Learning

**Figure 9:**
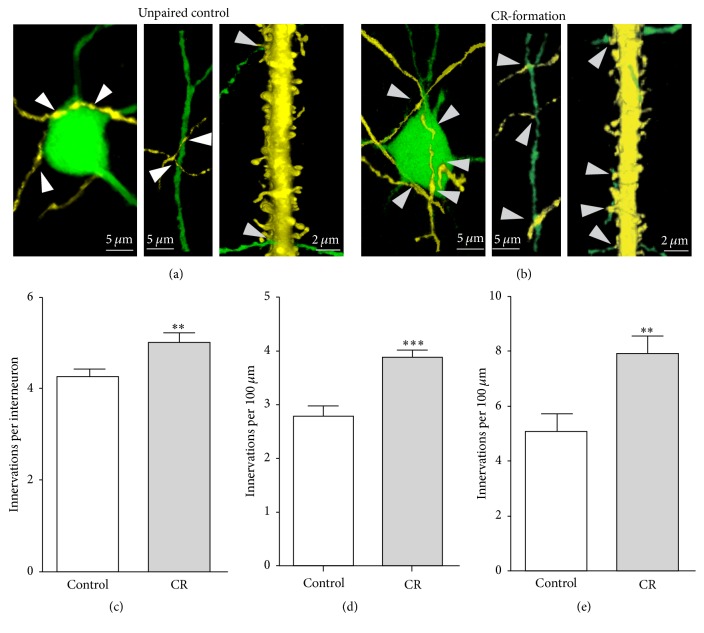
Mutual innervation between excitatory and inhibitory neurons is upregulated after associative learning. (a) shows YFP-labeled axon terminals on a GFP-labeled GABAergic neuron (left panel) and its process (middle) as well as GFP-labeled axon terminals on YFP-labeled apical dendrite of a glutamatergic neuron (right) from controls. (b) shows YFP-labeled axon terminals on a GFP-labeled GABAergic neuron (left panel) and its process (middle) as well as GFP-labeled axon terminals on YFP-labeled dendrite of a glutamatergic neuron (right) from CR-formation. White arrows indicate their termination. (c) shows YFP-labeled axon terminals on each GABAergic neuron in control (white bar) and CR-formation (gray, two asterisks, *p* < 0.01, *n* = 43). (d) shows YFP-labeled axon terminals per 100 *μ*m GFP-labeled dendrite of GABAergic neurons in controls (white bar) and CR-formations (gray, three asterisks, *p* < 0.001, *n* = 26). (e) shows GFP-labeled axon terminals per 100 *μ*m YFP-labeled apical dendrite of glutamatergic neurons in controls (white bar) and CR-formations (gray, two asterisks, *p* < 0.01, *n* = 19).

In addition to studying excitatory and inhibitory synapses, the interactions between glutamatergic and GABAergic neurons were investigated by counting YFP-labeled axon terminals on GFP-GABAergic neuron and GFP-labeled axon terminals on YFP-labeled apical dendrites of glutamatergic neuron (Figure 9). YFP-labeled axon terminals on the soma of GABAergic neuron are 4.26 ± 0.17 in controls (white bar in Figure 9(c)) and 5 ± 0.21 in CR-formations (gray, *p* < 0.01, *n* = 43). YFP-labeled axon terminals per 100 *μ*m dendrite from GABAergic neurons are 2.78 ± 0.2 in controls (white bar in Figure 9(d)) and 3.9 ± 0.15 in CR-formations (gray, *p* < 0.001, *n* = 26). GFP-labeled axon terminals per 100 *μ*m dendrite in the glutamatergic neuron are 5.11 ± 0.63 in controls (white bar in Figure 9(e)) and 7.91 ± 0.65 in CR-formations (gray, *p* < 0.01, *n* = 19). The mutual innervations between glutamatergic and GABAergic neurons are upregulated during associative memory.

### 3.5. The Changes of MicroRNAs Facilitate Neuron Recruitment and Refinement for Memory Formation

In terms of molecular mechanisms for the formation of new synapses as well as the recruitment of associative memory neurons in the barrel cortex, we assumed that coactivation of the cortices induced epigenetic changes, such as microRNA, which was examined by genome-wide sequencing to detect barrel cortical microRNA profile in CR-formation and control mice.  Table 1 shows the different expressions of microRNAs, in which some microRNAs decrease and others increase. Based on their gene targets (Table 1 referred to in the libraries https://www.ncbi.nlm.nih.gov/gene/ and http://www.targetscan.org/), the changed expression of microRNAs in CR-formation mice is associated with upregulating synapse formation, axon growth, and glutamatergic synapses, as well as downregulating GABA synapses. In addition to revealing new microRNAs involved in memory formation, the roles of microRNAs in regulating the neurons and synapses grant the indications about neuron-specific recruitment and refinement (Figures 2~9).

## 4. Discussion

**Figure 10:**
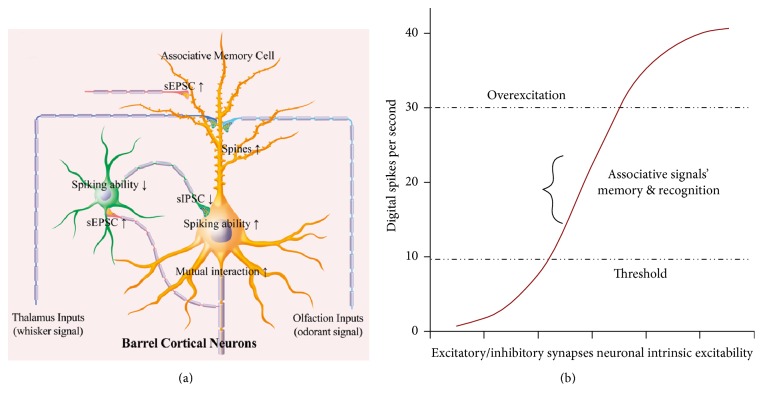
The coordinated recruitment and refinement of barrel cortical glutamatergic and GABAergic neurons set up their function state for information storage. (a) In addition to receiving whisker signal from the thalamus, associative memory cells in the barrel cortex receive odor signal from the piriform cortex after associative learning. In the glutamatergic neurons (orange), their dendritic spines are enriched, their excitatory synaptic transmissions are upregulated, and their receiving of inhibitory synaptic transmission is downregulated for their recruitments to be associative memory cells. The innervations from GABAergic axons are increased, such that the glutamatergic neurons are not overexcited. In the GABAergic neurons (green), their processes are enriched, their receptive fields of excitatory synaptic transmission are enhanced, and their innervation from the excitatory neurons is increased. Their synaptic outputs are decreased. The GABAergic neurons are homeostasis by coordinating their subcellular compartments. (b) An upregulation in the ratio of the excitatory synapses to the inhibitory synapses drives the digital spike encoding at the excitatory neurons over the threshold (out of “functional silence”) into an optimal state for the recruitment and refinement of associative memory cells. The extreme weakness of inhibitory synapses pushes these neurons to be overexcited for strong memory with no recognition. The curve of digital spikes is simulated based on our data, in which the normalized stimulations are integrated by the ratio of excitatory synapses to inhibitory synapses.

In mice that show odorant-induced whisker motion, glutamatergic and GABAergic neurons in the barrel cortices are recruited to be associative memory cells that encode new odor signal alongside innate whisker signal (Figure 1). In glutamatergic neurons, the increased excitatory synaptic input (Figures 2 and 3) and encoding ability (Figure 4) as well as the decreased inhibitory synaptic input (Figure 5) may facilitate their recruitments to be associative memory cells and drive them to optimal state for information storage (Figure 10). In GABAergic neurons, the upregulated excitatory synaptic input (Figures 6 and 7) promote their recruitment to be associative memory cells. The enhanced mutual innervations between glutamatergic and GABAergic neurons (Figure 9) maintain a homeostasis in local neural networks.

In terms of the allocation of information storage, the studies in withdrawal reflex [67–69], eyeblink-conditioning [70, 71] and fear-conditioning [72–74] suggest that the motor-related brain areas and neurons are critical. On the other hand, the sensory cortices are memory allocations [8, 28, 34]. These inconsistent conclusions can be explained by the possibility that memory presentation is fulfilled by the neuronal circuits from the sensory cortices to the motor cortices through their relays. This hypothesis is granted by the facts that stimulating any of these areas triggers memory retrieval [75–79] and the responses to the associated signals are detected in sensory cortices [28] and their downstream brain areas [30, 31, 80]. Thus, the sensory cortices are primary locations for information storage. Sensory cortical neurons that integrate associative signals for their storage can define signal specificity and expand memory volume.

In terms of plasticity at barrel cortical glutamatergic neurons, our study shows the upregulation of excitatory synapses and the downregulation of inhibitory synapses. Such changes make the glutamatergic neurons more excitable (Figure 10(b)), which permits excitatory driving force from the new synapse innervations of the piriform cortex [38] to recruit them as associative memory cells and to refine them with the upregulated ability to encode digital spikes [58, 81, 82] for memory formation. These associative memory cells and their upregulation boost their ability to activate the downstream neurons for behavior reactions and memory presentations during information retrievals. It is noteworthy that an increased inhibitory innervation and a decreased inhibitory synaptic transmission on the glutamatergic neurons maintain their functional homeostasis.

To plasticity at barrel cortical GABAergic neurons, our results indicate the upregulation of their excitatory synapses and receptive fields, which facilitate the recruitment of the GABAergic neurons to be associative memory cells. On the other hand, their synaptic outputs to inhibit target cells decrease, which facilitates the recruitment of other barrel cortical neurons for memory formation. This result is consistent with the fact that the disinhibition of neuronal circuits occurs during fear memory [34]. It is noteworthy that the increased excitatory input and the decreased inhibitory output in GABAergic neurons present another example about the maintenance of neuronal homeostasis by the coordination of different subcellular compartments [57], that is, neuronal homeostasis for memory formation.

In terms of recruiting associative memory cells and their coordination, we propose the following molecular and cellular processes. The coactivation of the barrel and piriform cortices induces epigenetic change (Table 1). The upregulated microRNAs knock down their target genes or vice versa. The altered expression of the genes and proteins facilitates axon prolongation, new synapse innervation, and excitatory synapse function as well as attenuates inhibitory synaptic function. These changes lead to the coordinated recruitment and refinement of the glutamatergic and GABAergic neurons to be associative memory cells. This assumption is granted by our current observation that anti-miRNA-324 and anti-miRNA-133a block associative memory and synapse innervation [38]. These consistent results by applying molecular, functional, and morphological approaches strengthen the conclusion reliability of our studies.

Whether the axons from the piriform cortex make the synapses on barrel cortical WS-responsive neurons or WS-nonresponsive neurons is based on the competition of these axons with the axons from the thalamus. If the axons from the piriform cortex competitively innervate WS-responsive cells in the barrel cortex, these WS-responsive neurons are recruited as associative memory cells that encode innate whisker signal and newly learnt odor signal. Otherwise, the axons from the piriform cortex turn to innervate WS-nonresponsive neurons in the barrel cortex to recruit them as new memory cells that encode odor signal only. Barrel cortical WS-responsive neurons that do not receive axon inputs from the piriform cortex still encode WS only. This hypothesis remains to be tested by dynamically observing synapse formation and axon growth in a neuron-specific manner.

Cognitive processes, such as logical reasoning, associative thinking, and comparison, require the associated retrievals of pair-stored signals from the distinct groups of associative memory cells. They may fulfill the retrievals of these pair-stored signals based on a pair-by-pair sequence of multiple-grades or the sharing of common signal in these pair-stored signals. In this regard, the newly wired axon circuits among different brain regions and the newly formed synapses in the circuits are essential to the communication of associative memory cells for the cognitions. Our studies reveal that mutual synapse innervation among the sensory cortices and associative memory cell recruitments [28, 29, 38] constitute the bases of associative memory and cognitive processes. The associative memory cells in the sensory cortices are recruited to encode the multiple associated signals. They receive multiple synapse innervations from the associatively activated sensory areas that encode their respective signals. Their axons project to the associatively activated sensory cortices for their mutual innervations as well as to the cognitive, emotional, and behavioral cortices for memory presentation. The recruitments of synapse innervation and associative memory cells are upregulated or downregulated by changing the gene expressions through miRNA manipulation, which influences memory capacity.

## Supplementary Material

A simultaneous pairing of WS and OS leads to odorant-induced whisker motion.

## Figures and Tables

**Table 1:** MicroRNAs' expression and their role in associative learning.

Name of microRNA	The microRNA expressions of CR-formation versus naïve control in the barrel cortex	Predicted target genes	Functions of target genes (+), upregulation (−), downregulation	Effects of microRNA expression changes on CR-formation
mmu-miR-5128	↓	Fgfr2 Neto1	Excitatory synapses (+)	Upregulate excitatory synapses

mmu-miR-342-5p	↓	Elfn1 Fgfr2 Gria1 Grin1 Lrrtm2 Nlgn3	Excitatory synapses (+)	Upregulate excitatory synapses

mmu-miR-342-5p	↓	Nrxn1 Nlgn3	Synapse formation (+)	Upregulate synapse formation

mmu-miR-150-5p	↑	Nlgn2 Csnk1d	Excitatory synapses (−) Neuron outgrowth (−)	Upregulate excitatory synapses Upregulate neuron outgrowth

mmu-miR-3072-3p	↑	Wnk2	Channels (+/−)	Upregulate excitatory synapses

mmu-miR-3072-3p	↑	Slc32a1	Inhibitory synapses (+)	Downregulate inhibitory synapses

mmu-miR-150-5p	↑	Nlgn2	Inhibitory synapses (+)	Downregulate inhibitory synapses

mmu-miR-324-5p	↑	Slc32a1	Inhibitory synapses (+)	Downregulate inhibitory synapses

mmu-miR-23b-3p	↑	Nrxn1	Inhibitory synapses (+)	Downregulate inhibitory synapses

mmu-miR-133a-3p	↑	Iqsec3	Inhibitory synapses (+)	Downregulate inhibitory synapses

mmu-miR-345-5p	↑	Gabra4	Inhibitory synapses (+)	Downregulate inhibitory synapses

mmu-miR-345-5p	↑	Rgma	Synapse formation or neuron branching (−) Spines and dendrites (−)	Upregulate synapse formation or neuron branching Stabilize spines and dendrites

mmu-miR-324-5p	↑	Ttbk1	Spines and dendrites (−) Axon guidance (−)	Stabilize spines and dendrites Upregulate axon growth and branching

mmu-miR-3072-3p	↑	Mark2	Axon guidance (−)	Upregulate axon growth and branching

mmu-miR-133a-3p	↑	Dyrk2	Axon guidance (−) Cytoskeleton (−) Neurite outgrowth (−) Neuron proliferation (−)	Upregulate axon growth and branching Stabilize neuron morphology Upregulate dendrite growth Upregulate neuron proliferation
