# Supplementary material for: Coordinated Plasticity between Barrel Cortical Glutamatergic and GABAergic Neurons during Associative Memory
Source: Neural Plast. 2016 Dec 14;2016:5648390. doi: 10.1155/2016/5648390 (PMC5192352; doi:10.1155/2016/5648390)

Figure S1 A simultaneous pairing of WS and OS leads to odorant-induced whisker motion. The odor-test is butyl acetate pulse (20 seconds) toward the noses and the recordings of whisker motion by a digital video camera are digitized into motion traces. **A)** shows the mouse whisker motion in response to odor-test pulses (top traces) before (middle traces) versus after training (bottom traces) in WS/OS-paired mice (left panel) and WS/OS-unpaired. Calibration bars are thirty degrees of whisker retraction and 5 seconds. **B~C)** show whisking frequency (**B**) and retraction duration (**C**) in response to the odor-test in the groups of WS/OS-paired mice and WS/OS-unpaired before (blue bars) and after trainings (red). The significant changes of such parameters are seen in WS/OS-paired mice, i.e., odorant-induced whisker motion (n=14, p<0.001; paired t-test for comparisons before versus after training).

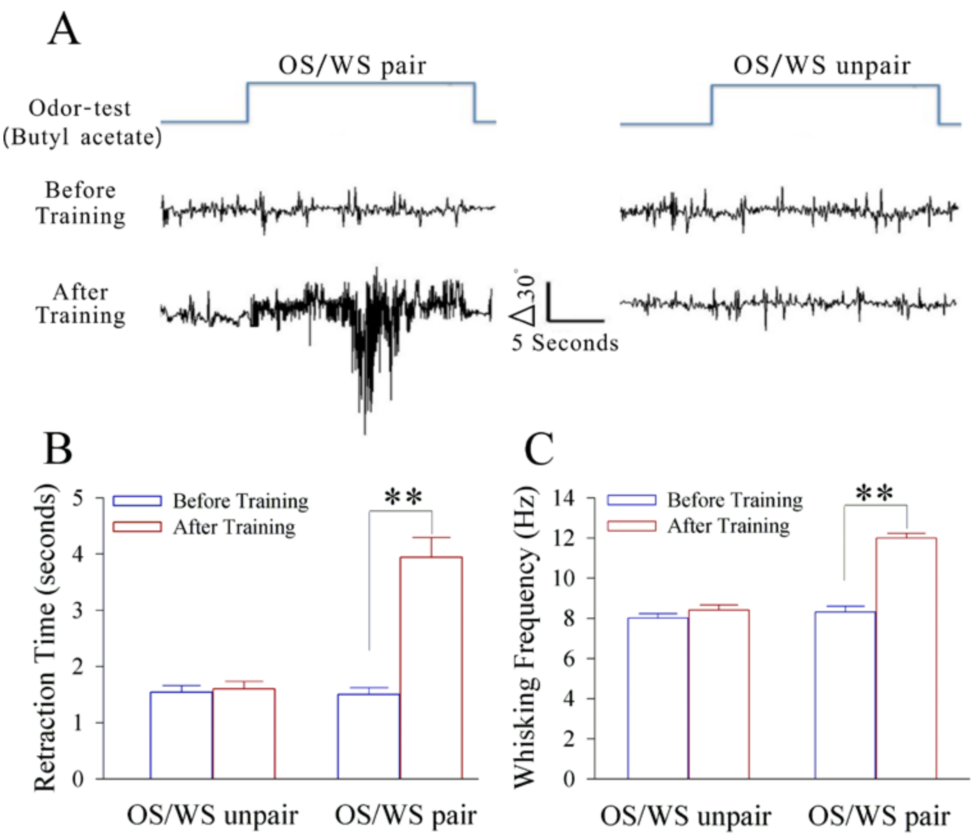

Supplement: Supplementary file 1 — A simultaneous pairing of WS and OS leads to odorant-induced whisker motion. [file 5648390.f1.pdf]
